# Role of Alkyl Chain Length in Surfactant-Induced Precipitation of Reactive Brilliant Blue KN-R

**DOI:** 10.3390/molecules29030619

**Published:** 2024-01-28

**Authors:** Hongyu Liu, Yunkang Chang, Yuhuan Li, Chengsong Cao, Rui Li

**Affiliations:** School of Biological Science, Jining Medical University, No. 669 Xueyuan Road, Donggang District, Rizhao 276826, China; liuhongyu@mail.jnmc.edu.cn (H.L.); 17863326811@163.com (Y.L.); 15955134844@163.com (C.C.)

**Keywords:** dye–surfactant interaction, alkyl chain length, precipitation, reactive brilliant blue KN-R

## Abstract

To develop a cost-effective method for the effective removal of reactive brilliant blue KN-R (RBB KN-R) from wastewater, we investigated the interactions between RBB KN-R and three cationic surfactants with different alkyl chain lengths, namely dodecyltrimethylammonium bromide (DTAB), tetradecyltrimethylammonium bromide (TTAB), and cetyltrimethylammonium bromide (CTAB). Employing a conductivity analysis, surface tension analysis, ultraviolet-visible spectrophotometry, and molecular dynamics simulation, we ascertained that RBB KN-R formed a 1:1 molar ratio dye–surfactant complex with each surfactant through electrostatic attraction. Notably, an augmentation in alkyl chain length correlated with increased binding strength between RBB KN-R and the surfactant. The resulting dye–surfactant complex exhibited heightened surface activity, enabling interactions through hydrophobic forces to generate dye–surfactant aggregates when the molar ratio was below 1:1. Within these mixed aggregates, self-assembly of RBB KN-R molecules occurred, leading to the formation of dye aggregates. Due to the improved hydrophobicity with increased alkyl chain length, TTAB and CTAB could encapsulate dye aggregates within the mixed aggregates, but DTAB could not. The RBB KN-R aggregates tended to distribute on the surface of the RBB KN-R-DTAB mixed aggregates, resulting in low stability. Thus, at a DTAB concentration lower than CMC, insoluble particles readily formed and separated from surfactant aggregates at an RBB KN-R and DTAB molar ratio of 1:4. Analyzing the RBB KN-R precipitate through scanning electron microscopy (SEM) and Fourier transform infrared spectroscopy (FTIR) and measuring the DTAB concentration in the supernate revealed that, at this molar ratio, all RBB KN-R precipitated from the dye–surfactant mixed solution, with only 7.5 ± 0.5% of DTAB present in the precipitate. Furthermore, the removal ratio of RBB KN-R reached nearly 100% within a pH range of 1.0 to 9.0 and standing time of 6 h. The salt type and concentration did not significantly affect the precipitation process. Therefore, this simultaneous achievement of successful RBB KN-R removal and effective separation from DTAB underscores the efficacy of the proposed approach.

## 1. Introduction

In recent decades, the discharge of dye-containing wastewater into the environment has witnessed a disturbing surge due to the rapid growth of various industries [1]. Among these dyes, the synthetic, water-soluble, and non-biodegradable reactive brilliant blue KN-R (RBB KN-R) has attracted significant attention, owing to its extensive usage in the textile, paper, and leather industries [2,3,4]. The release of this dye into natural water bodies poses a grave threat to aquatic life and human health [5,6,7]. Thus, the removal of RBB KN-R from the aqueous phase assumes paramount importance.

Adsorption and membrane separation have been extensively documented as effective methods for the removal of RBB KN-R [8,9,10,11]. Despite their high separation efficiency, both the techniques have high operation costs and complicated processes [12,13]. Dyes exhibit favorable interactions with surfactants through electrostatic and hydrophobic forces, enabling their removal from wastewater using low-cost separation techniques [14,15,16]. For instance, Melo et al. [17] employed carboxylate surfactants to enhance the solubilization of Reactive Blue 14 within micelles, followed by the addition of calcium ions to induce micelle flocculation. They achieved an impressive dye removal rate of 86%. A similar methodology was employed by Sultana et al. [18] for the efficient elimination of reactive yellow 160. In both studies, surfactants played a crucial role in enhancing dye solubilization, while the use of flocculants was also necessary. Mahbub et al. [19] also made a noteworthy observation that in the presence of salt and ethanol, the combination of reactive red and cetyl trimethylammonium bromide (CTAB) formed mixtures that readily precipitated to the bottom of the water. Based on these papers in the literature, we attempt to explore the application of surfactant-induced precipitation for the removal of RBB KN-R from wastewater.

In Refs. [17,18,19], the elucidation of interactions between the chosen surfactant and dye molecules were well-documented. However, a comprehensive explanation of the specific surfactant characteristics conducive to dye precipitation is notably absent. Furthermore, all the used surfactant concentrations were higher than their critical micelle concentrations (CMCs). Prior studies by Abe et al. [20], Rashidi-Alavijeh et al. [21], and Ali et al. [22] found that the alkyl chain length of a surfactant played a pivotal role in the surfactant–dye interactions by varying the micellar structures. In specific, the increase in alkyl chain length enhanced the dye–surfactant interaction and also enlarged the size of dye–surfactant mixed aggregates [20,21,22]. In this case, the surfactant with the longer alkyl chain seems to have a better ability to precipitate RBB KN-R. Nevertheless, an examination of the influence of alkyl chain length on surfactant-induced dye precipitation remains unexplored. In light of these considerations, our investigation endeavors to scrutinize the impact of the alkyl chain length of a surfactant on the precipitation of RBB KN-R.

**Figure 1:**
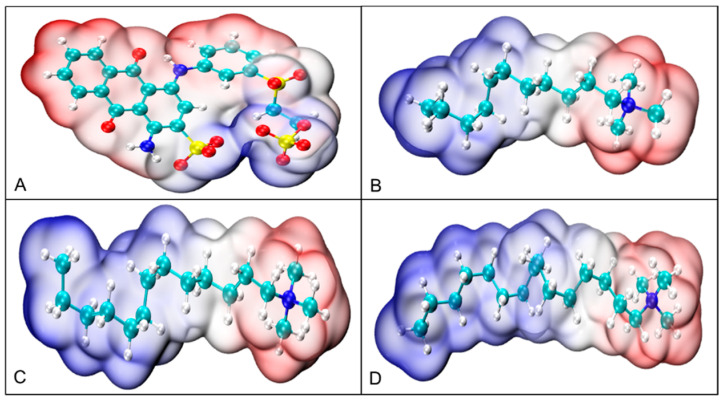
Structures of RBB KN-R (**A**), DTAB (**B**), TTAB (**C**), and CTAB (**D**).

Given that RBB KN-R is an anionic dye, we have chosen three commonly used quaternary ammonium surfactants—dodecyltrimethylammonium bromide (DTAB), tetradecyltrimethylammonium bromide (TTAB), and CTAB—to induce its precipitation. The structures of these four chemicals are depicted in Figure 1. Our investigation into the effect of alkyl chain length on the precipitation of RBB KN-R will focus on three key aspects: (1) experimental studies on the impact of alkyl chain length on the dye–surfactant interactions using conductivity analysis, surface tension analysis, and ultraviolet-visible (UV-Vis) spectrophotometry [19], (2) molecular dynamics (MD) simulation of the impact of alkyl chain length on the dye–surfactant interactions [19,21], (3) experimental confirmation of the separation of dye aggregates from surfactants by characterizing the dye precipitates through scanning electron microscopy (SEM) and Fourier transform infrared spectroscopy (FTIR), and (4) optimization of removal of RBB KN-R using DTAB-induced precipitation.

## 2. Results and Discussion

### 2.1. Effects of Alkyl Chain Length on Conductivity of Surfactant Solution with and without RBB KN-R

**Figure 2:**
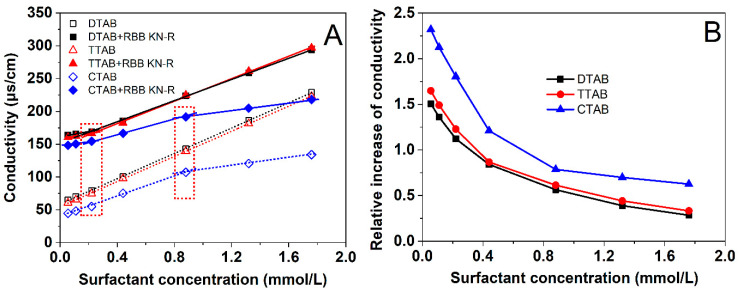
Dependencies of solution conductivity and on DTAB, TTAB, and CTAB concentrations with and without RBB KN-R (**A**) and dependencies of relative increase in conductivity due to RBB KN-R on DTAB, TTAB, and CTAB concentrations (**B**).

In an aqueous solution, the interaction between an ionic dye and a surfactant of opposite electrical charge can be attributed to electrostatic attraction, resulting in a noticeable modification of the solution’s conductivity [23,24,25]. Thus, we investigated the electrostatic interactions between RBB KN-R and three surfactants, namely DTAB, TTAB, and CTAB, by monitoring the changes in solution conductivity corresponding to varying surfactant concentrations. The results are presented in Figure 2.

The outcomes depicted in Figure 2A illustrate a linear increase in solution conductivity for DTAB and TTAB, ranging from 0.055 mmol/L to 1.76 mmol/L, in the absence of RBB KN-R. Such an observation implies that the CMCs for both surfactants exceeded 1.76 mmol/L in the 0.2 mmol/L Na_2_HPO_4_ solution of 7.0 [26,27]. In contrast, CTAB exhibited a faster linear rise in solution conductivity between 0.055 mmol/L and 0.88 mmol/L compared to the range of 0.88 mmol/L to 1.76 mmol/L. These results suggest that the CMC of CTAB approximated 0.88 mmol/L [27]. Upon the introduction of RBB KN-R into each surfactant solution, a substantial increase in solution conductivity was observed due to the dye dissociation. Notably, the relationship between solution conductivity and surfactant concentration remained linear. The slope for the higher concentration range (>0.22 mmol/L) was identical to that observed without the presence of RBB KN-R, albeit it was greater than the slope for the lower concentration range (<0.22 mmol/L). These findings suggest that RBB KN-R interacted with each surfactant in a 1:1 molar ratio under the prevailing solution conditions [24,26].

The solution conductivity was essentially determined by ion concentration [28]. Figure 2A illustrates that in the absence of RBB KN-R, the conductivity of the DTAB solution closely approximated that of the TTAB solution but surpassed that of the CTAB solution. This observation suggests that CTAB exhibited a diminished ionization level compared to both DTAB and TTAB within the given solvent. Conversely, as depicted in Figure 2B, the introduction of RBB KN-R led to a higher relative increase in conductivity for CTAB compared to TTAB. Meanwhile, CTAB experienced a slightly greater relative increase than DTAB at each surfactant concentration. These findings imply a hierarchy in the relative enhancements of ionization levels induced by RBB KN-R, with the order being CTAB > TTAB > DTAB. This order corresponds to the strength of electrostatic interactions between RBB KN-R and the respective surfactants, specifically RBB KN-R-CTAB > RBB KN-R-TTAB > RBB KN-R-DTAB.

### 2.2. Effects of Alkyl Chain Length on Surface Tension of the Surfactant Solution with and without RBB KN-R

**Figure 3:**
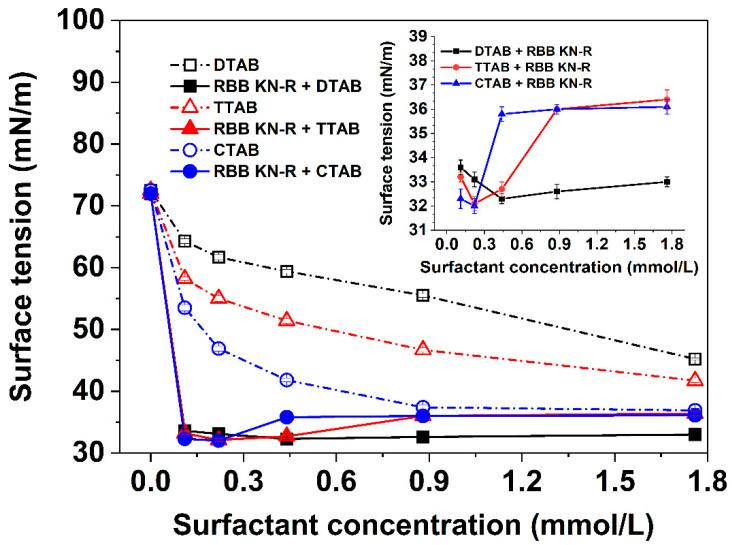
Dependencies of surface tension on DTAB, TTAB, and CTAB concentrations with and without RBB KN-R.

In this section, the interactions between RBB KN-R and DTAB, TTAB, and CTAB were further investigated by comparing the variations in surface tension with surfactant concentration in the presence and absence of RBB KN-R. The experimental results depicted in Figure 3 demonstrate that, in the absence of RBB KN-R, the surface tensions of the DTAB and CTAB solutions exhibited a gradual decrease as the surfactant concentration increased. However, the surface tension of the CTAB solution initially decreased and then reached a constant value as the CTAB concentration increased from 0 to 0.88 mmol/L to 1.76 mmol/L. These findings suggest that the CMC of DTAB and TTAB exceeded 1.76 mmol/L, while the CMC of CTAB was approximately 0.88 mmol/L. These results corroborated the observations presented in Figure 2A. Additionally, at a fixed concentration, the surface tensions of the three surfactants followed the order of DTAB > TTAB > CTAB, indicating that their surface activities ranked as CTAB > TTAB > DTAB. Consequently, the enhancement of alkyl chain length positively influenced the surface activity of the surfactant, in agreement with the previous studies of Sastry et al. [29] and Verma et al. [30].

It is also observed from Figure 3 that, for each surfactant, the presence of RBB KN-R led to a significant decrease in surface tension compared to samples without RBB KN-R. The surface tension gradually decreased as the concentration of surfactant increased from 0 to 0.22 mmol/L. This finding indicates that the complex formed by RBB KN-R and the surfactant had higher surface activity than the surfactant alone. In the absence of surfactant (surfactant concentration = 0), the surface tension of the RBB KN-R solution was equivalent to that of the solvent, suggesting a very weak surface activity of RBB KN-R. However, the electrostatic interaction between RBB KN-R and the surfactant reduced the polarity of the surfactant and introduced hydrophobic groups from RBB KN-R [21,31]. Consequently, the dye–surfactant complex exhibited enhanced surface activity. At lower surfactant concentrations (<0.22 mmol/L), the surface tensions of the three dye–surfactant solutions followed the order of RBB KN-R-DTAB solution < RBB KN-R-TTAB solution < RBB KN-R-CTAB solution, consistent with the surface tensions of the respective surfactants alone.

Upon saturation of all RBB KN-R molecules by surfactant molecules at a concentration of 0.22 mmol/L, a subsequent increase in surfactant concentration resulted in elevated surface tension within the dye–surfactant solution. This phenomenon resulted from the displacement of dye–surfactant complexes at the air–water interface by free surfactant molecules [32]. The increased concentration of free surfactant molecules facilitated their interaction with dye–surfactant complexes, leading to the formation of more hydrophilic aggregates with the hydrophobic complexes situated internally. Interestingly, the rate of surface tension increase in the dye–surfactant solution correlated positively with the alkyl chain length. Specifically, at a surfactant concentration of 1.76 mmol/L, the surface tension of the RBB KN-R-CTAB solution was just slightly different to that of the CTAB solution. This surface tension displayed no significant difference from that of the RBB KN-R-TTAB solution but surpassed that of the RBB KN-R-DTAB solution. The observation implies that surfactants with longer alkyl chains more effectively encapsulate hydrophobic dye–surfactant complexes within the interior of hydrophilic dye–surfactant aggregates due to their higher surface activity [33,34].

### 2.3. Effect of Alkyl Chain Length on UV-Vis Spectra of the Dye–Surfactant Solution

**Figure 4:**
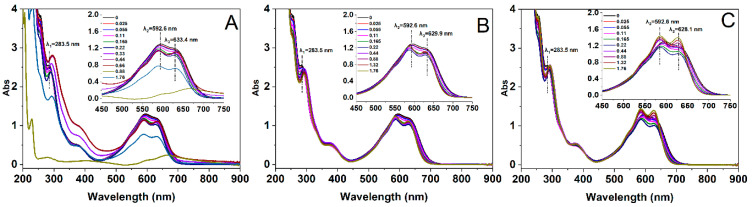
UV-Vis spectra of RBB KN-R at DTAB (**A**), TTAB (**B**), and CTAB (**C**) concentrations from 0 to 1.76 mmol/L.

UV-Vis spectrophotometry is a widely employed technique for investigating the interaction between dyes and surfactants [35]. In this specific study, we utilized both methods to assess the interaction between RBB KN-R and DTAB, TTAB, as well as CTAB and its aggregation. The results are presented in Figure 4.

Figure 4A demonstrates that the UV-Vis spectrum of RBB KN-R exhibited two distinct adsorption peaks at 592.6 nm and 283.5 nm, corresponding to the chromophoric and aromatic groups, respectively. With the incremental addition of DTAB into the RBB KN-R solution to 0.22 mmol/L, the peak at 592.6 nm underwent a progressive blue shift, while the peak at 283.5 nm showed a red shift, accompanied by a decrease in their respective absorbance values. Simultaneously, a novel peak emerged at 633.4 nm at 0.22 mmol/L. In Figure 4B,C, similar phenomena were also observed for TTAB and CTAB in the surfactant range from 0 to 0.22 mmol/L. Based on the results in Figure 2, the RBB KN-R molecules gradually interacted with the surfactant molecules to form ion pairs at a molar ratio of 1:1 with the successive addition of surfactant up to 0.22 mmol/L. The dye–surfactant complexes had high hydrophobic properties (Figure 3), so their aggregates were readily formed via hydrophobic forces [36,37], corresponding to the appearance of peaks at approximately 630 nm. As a result, the UV-Vis absorption intensity of each dye–surfactant solution gradually decreased as the surfactant concentration increased to 0.22 mmol/L. The blue shift of the peak at 592.6 nm was attributed to the formation of RBB KN-R aggregates in a face-to-face arrangement (H-aggregation) within the aggregated dye–surfactant ion pairs, while the red shift of the peak at 283.5 nm was a consequence of the Π-Π interaction between aromatic rings in the dye aggregates [19,38]. Furthermore, Figure 4 presents that at a surfactant concentration of 0.22 mmol/L, the peaks corresponding to the aggregated dye–surfactant complexes were observed at 633.4 nm for DTAB, 629.9 nm for TTAB, and 628.1 nm for CTAB. This finding suggests a progressive enhancement in the hydrophobicity of the microenvironment surrounding the dye–surfactant aggregates, with an elongation of the alkyl chain length.

**Figure 5:**
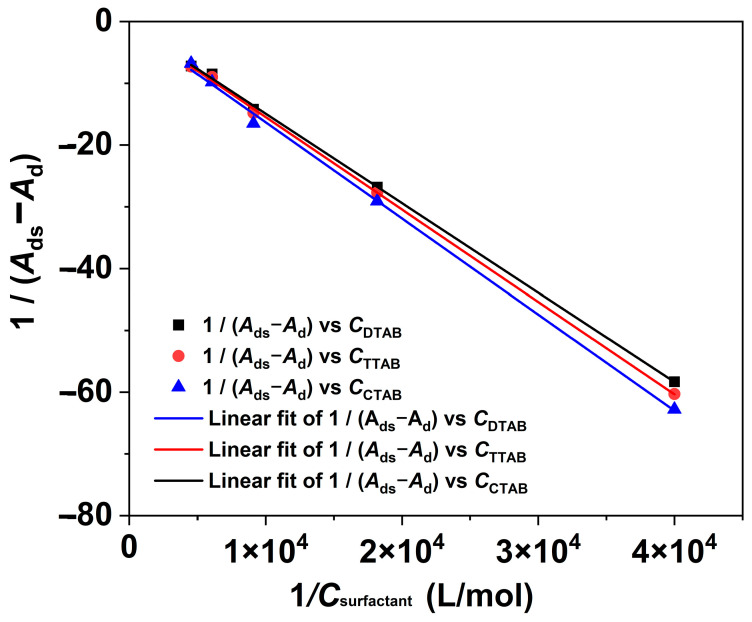
The Benesi–Hildebrand plots for RBB KN-R-DTAB, RBB KN-R-TTAB, and RBB KN-R-CTAB systems.

Based on the above analysis, the Benesi–Hildebrand equation, as described in Equation (1) [39], was further used to calculate the binding constants (*k*_b_) for the interactions between RBB KN-R and the DTAB, TTAB, and CTAB surfactants.

(1) $$1/(A_{\mathrm{ds}}-A_{d})=1/((A_{i}-A_{d})k_{b}C_{\mathrm{surfactant}})+1/(A_{i}-A_{d}).$$

where *A*_d_ and *A*_ds_ denote the absorbance of RBB KN-R with and without a surfactant, respectively, *A*_i_ represents the absorbance at the infinite concentration caused by the dye–surfactant ion pair formation, and *C*_surfactant_ stands for the surfactant concentration ranging from 0 to 0.22 mmol/L. The results obtained from the fitting process are presented in Figure 5 and Table 1. The fitting results demonstrate significant outcomes, with 1/(*A*_ds_ − *A*_d_) and 1/*C*_surfactant_ exhibiting strong linear relationships for each surfactant. It is indicated that each surfactant interacted with RBB KN-R at the molar ratio of 1:1 [19]. Based on these findings, the *k*_b_ values for the interaction between the dye and surfactant were calculated. As shown in Table 1, it was observed that the *k*_b_ value increased with an increase in the alkyl chain length. These findings were consistent with those in Figure 2.

In Figure 4A, as the concentration of DTAB increased from 0.22 mmol/L to 0.66 mmol/L, both the peaks corresponding to the chromophoric groups and the dye–surfactant aggregates suffered a red shift, accompanied by a gradual rise in absorbance. This phenomenon signifies that the sequential addition of DTAB led to an augmentation in the H-aggregation of RBB KN-R and the exposure of the dye aggregates to a more hydrophilic microenvironment [40]. Upon further elevating the DTAB concentration to 0.88 mmol/L, the aggregation of the dye was notably intensified. This resulted in the generation of insoluble particles and a marked reduction in UV-Vis adsorption intensity of the RBB KN-R-DTAB solution (Figure 4A). Nevertheless, when the DTAB concentration surpassed 0.88 mmol/L, some of the insoluble dye aggregates underwent re-dissolution in water, giving rise to RBB KN-R-DTAB micelles characterized by internally hydrophobic and externally hydrophilic structures [41]. Consequently, the UV-Vis adsorption intensity of the dye–surfactant solution experienced enhancement. From Figure 4B,C, as the concentrations of TTAB and DTAB increased from 0.22 mmol/L to 1.76 mmol/L, the absorption peaks of the chromophoric groups (586.5 nm for DTAB and 585.9 nm for CTAB at 0.22 mmol/L) underwent further blue shifts, while their absorbance gradually increased. On the other hand, the peaks corresponding to the aromatic groups (292.5 nm for DTAB, CTAB at 0.22 mmol/L) did not exhibit significant shifts, with their absorbance gradually decreasing. Meanwhile, the adsorption intensities of the peaks pertaining to the dye–surfactant aggregates (629.9 nm for DTAB and 628.1 nm for CTAB at 0.22 mmol/L) also suffered positive increments with respect to surfactant concentration. These findings establish that higher concentrations of TTAB and CTAB allowed for a greater number of surfactant molecules to surround the RBB KN-R surfactant complexes to take them into a more hydrophobic microenvironment. This did not cause insoluble aggregation of RBB KN-R. Furthermore, CTAB induced more pronounced increases in absorbance for both the chromophoric and aromatic groups than TTAB across the surfactant concentration range of 0.22 mmol/L to 1.76 mmol/L. Thus, the increase in the alkyl chain length resulted in a higher level dissolution of the dye–surfactant complex in water.

### 2.4. MD Simulations of Interactions of RBB KN-R with DTAB, TTAB, and CTAB

**Figure 6:**
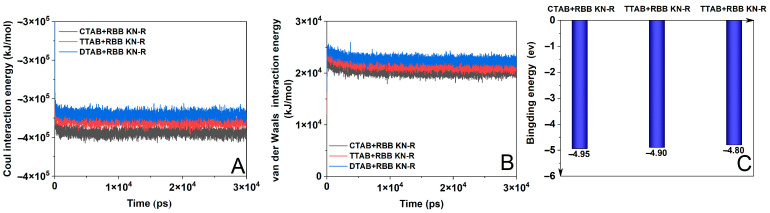
Coulomb interaction energy (**A**), van der Waals interaction energy (**B**), and binding energy (**C**) for RBB KN-R-DTAB, RBB KN-R-TTAB, and RBB KN-R-CTAB systems.

**Figure 7:**
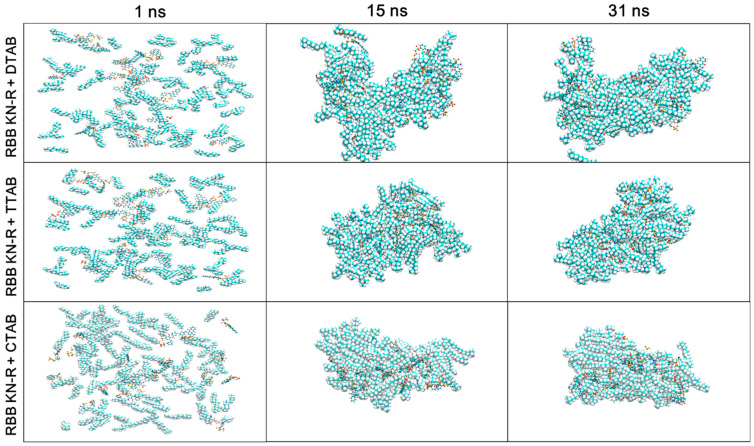
Images of MD trajectories of RBB KN-R-DTAB, RBB KN-R-TTAB, and RBB KN-R-CTAB systems at 1, 15, and 30 ns.

MD simulation is a classic method to gain a better understanding of the dye–surfactant interaction [42]. Thus, we used this method to analyze the interactions of RBB KN-R with DTAB, TTAB, and CTAB. The results are presented in Figure 6 and Figure 7.

In Figure 6A, a discernible increase in energy for the coulombic interaction between the dye and surfactant is observed as the alkyl chain length decreases. Because a lower energy often corresponds to a stronger interaction [43,44], this result indicates that RBB KN-R demonstrates the highest degree of electrostatic interaction with CTAB, an intermediate level with TTAB, and the lowest level with DTAB. Furthermore, Figure 6B illustrates a parallel trend in the variation in van der Waals interaction energy with alkyl chain length compared to coulombic interaction energy. However, the van der Waals interaction energy surpassed the coulombic interaction energy in magnitude for each instance of dye–surfactant interaction. This outcome underscores that electrostatic attraction was much stronger than van der Waals forces, thus playing a critical role in mediating interactions between RBB KN-R and the surfactants. In this case, the variation in binding energy for the dye–surfactant with the alkyl chain length in Figure 6C was consistent with that of the coulomb interaction energy in Figure 6A. In summary, the above results of the MD simulation confirm the experimental ones in Figure 2 and Table 1.

Figure 7 depicts the MD trajectories of the RBB KN-R-DTAB, RBB KN-R-TTAB, and RBB KN-R-CTAB systems at time points of 1, 15, and 30 ns. The visual representation illustrates the gradual aggregation of dye–surfactant complexes and free surfactant molecules over time, primarily driven by hydrophobic forces. Concurrently, there is a progressive interaction among the dye molecules themselves. Notably, in the case of RBB KN-R, a significant portion of the aggregates is found on the surface of the DTAB aggregates, while being entrapped within the aggregates of TTAB and CTAB. The stability of dye aggregates on the surfactant aggregates’ surface was lower compared to those within the interior. Consequently, these surface aggregates may readily form insoluble precipitates, leading to their separation from DTAB aggregates due to weaker dye–surfactant interactions. These observations were consistent with the analyses derived from Figure 4. It was previously reported that dyes have a stronger interaction with surfactants with longer alkyl chains [29,33]. On this ground, more researchers tended to use surfactants with higher surface activities to interact with dye molecules to form mixed micelles with larger sizes and then remove them by flocculation via varying solution conditions or membrane separation [17,18,19,45]. However, a very different finding in the current work was that RBB KN-R was directly precipitated by a surfactant with lower surface activity (DTAB). In comparison, the DTAB-induced precipitation has lower costs and simpler operation.

### 2.5. Characterization of RBB KN-R Precipitate

#### 2.5.1. SEM

**Figure 8:**
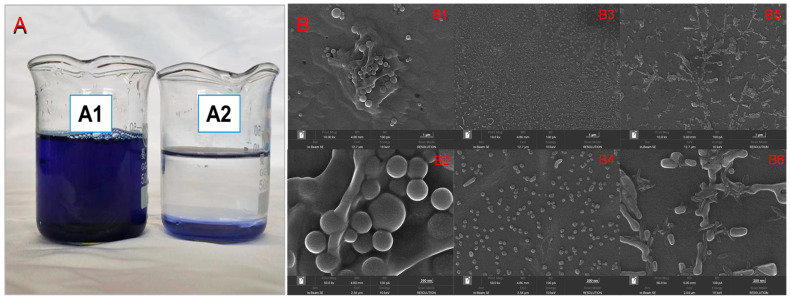
Optical photographs (**A**) of mixed solution of RBB KN-R (0.22 mmol/L) and DTAB (0.88 mmol/L) standing for 1 h (**A1**) and 8 h (**A2**), and SEM images (**B**) of DTAB (**B1** amplification ×10,000, **B2** amplification ×50,000), RBB KN-R (**B3** amplification ×10,000, **B4** amplification ×50,000), and RBB KN-R precipitate (**B5** amplification ×10,000, **B6** amplification ×50,000).

Based on the outcomes detailed in Section 3.3 and Section 3.4, it has been ascertained that DTAB exhibits the capability to induce the insoluble aggregation of RBB KN-R. This phenomenon has been directly substantiated through visual evidence captured in optical representations, as depicted in Figure 8A. It is illustrated that 100% of the RBB KN-R was precipitated at a RBB KN-R vs. DTAB molar ratio of 1:4 after the dye–surfactant solution stood for 8 h, corresponding to a DTAB concentration of 0.88 mmol/L, which is lower than its CMC. After centrifugation and drying, the morphology of the precipitate was scanned by a scanning electron microscope (SEM, MIRA LMS, TESCAN, Czech Republic). The SEM images in Figure 8B shows that the irregular shapes of the precipitate particles were very different from those of RBB KN-R and DTAB. In addition, they had lager sizes than the RBB KN-R particles but smaller sizes than DTAB particles. These results indicate that the RBB KN-R aggregates were the major ingredient in the precipitate.

#### 2.5.2. FTIR

**Figure 9:**
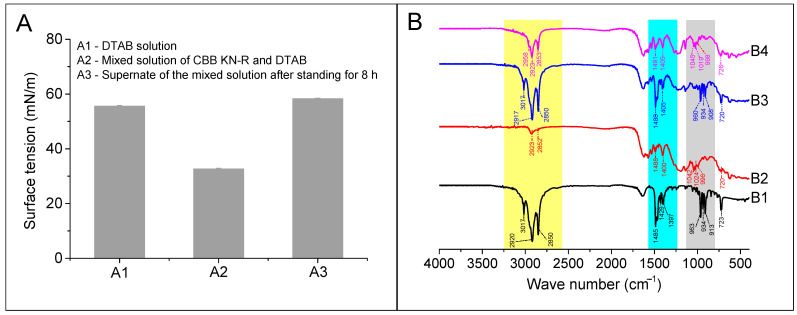
Surface tensions (**A**) of DTAB solution at 0.88 mmol/L (A1), mixed solution of RBB KN-R (0.22 mmol/L) and DTAB (0.88 mmol/L) after standing for 1 h (A2), and supernate of mixed solution of RBB KN-R (0.22 mmol/L) and DTAB (0.88 mmol/L) after standing for 8 h (A3), and FTIR spectra (**B**) of DTAB (B1), RBB KN-R (B2), physical mixture of DTAB and RBB KN-R at a molar ratio of 1:1 (B3), and precipitate obtained from A3 (B4).

In this section, a comparative assessment of the surface tension of the RBB KN-R (0.22 mmol/L)–DTAB (0.88 mmol/L) solution before and after the precipitation was first undertaken. Then, scrutiny of the FTIR spectra corresponding to the precipitates and the physically combined mixture of DTAB and RBB KN-R, upheld at a 1:1 molar ratio, was conducted. This was conducted in order to give a more comprehensive analysis of the process governing the formation of these precipitates. A detailed exposition of these findings is provided in Figure 9A,B, respectively. Figure 9A shows that the addition of RBB KN-R to the DTAB solution caused a significant decrease in surface tension, which was attributed to the interaction between the dye and surfactant. It is noteworthy that the surface tension of the supernatant improved and was slightly higher than that of the DTAB solution after RBB KN-R was removed. These results suggest that only a small number of DTAB molecules participated in the insoluble aggregation of RBB KN-R. Furthermore, we used the method for measuring the CTAB concentration reported by Kumar et al. [46] to detect the DTAB concentration in the supernatant. Then, the molar ratio of RBB KN-R vs. DTAB in the precipitate was calculated as 1: 0.14 (±0.01), corresponding to a DTAB mass ratio of 7.5 ± 0.5%.

From Figure 1, it is clear that –CH_2_ is the group with the largest content in DTAB. As expected, its characteristic peaks at 3017, 2920, 2850, 1485, and 723 cm^−1^ were easily visible in the FTIR spectrum of DTAB, as shown in Figure 9B. These peaks were also present in the FTIR spectra of RBB KN-R, the physical mixture of RBB KN-R and DTAB, and the precipitate, with the area of these peaks following the order: physical mixture of RBB KN-R and DTAB > the precipitate > RBB KN-R. In addition, the characteristic peaks at 963, 934, and 913 cm^−1^ denoted the presence of –CH groups in olefin ring compounds, which were attributed to impurities present in the DTAB sample. Despite this, these peaks were clearly present in the spectra of DTAB and the physical mixture but were not found in the spectrum of the precipitate. The characteristic peaks at 1400, 1042, 1024, and 996 cm^−1^ in the FTIR spectrum of RBB KN-R represent –CH groups in aromatic rings. These peaks were also found in the spectrum of the precipitate but were masked by the peaks for impurities in the DTAB sample in the FTIR spectrum of the physical mixture. Based on these findings, it is suggested that RBB KN-R exists in the precipitate. Overall, these results indicate that the molar ratio of RBB KN-R and DTAB in the precipitate was much higher than 1:1, corresponding to a small amount of DTAB, thus confirming the conjecture from Figure 7 and the results in Figure 8B and Figure 9A. In addition, the DTAB in the supernatant could be recovered by foam fractionation for reuse [46,47,48].

### 2.6. Optimization of Removal of RBB KN-R Using DTAB-Induced Precipitation

#### 2.6.1. Effect of pH on the Removal Ratio of RBB KN-R

**Figure 10:**
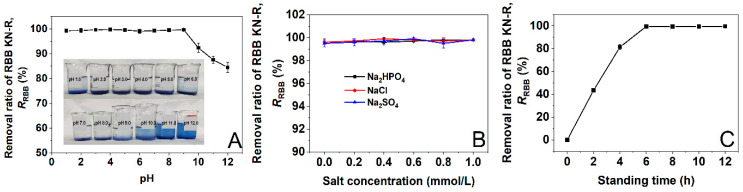
Effects of pH (A), salt type and concentration (**B**), and standing time (**C**) on the removal ratio of RBB KN-R.

The results presented in Section 3.1 and Section 3.2 demonstrated that DTAB was able to interact with RBB KN-R to form insoluble aggregates at a dye–surfactant molar ratio of 1:4. We then attempted to use DTAB to remove RBB KN-R from its simulated wastewater. In this section, we investigated the effect of pH on the removal rate of RBB KN-R as pH has significant effects on the dye–surfactant interaction and dye aggregation [14]. The wastewater was prepared by dissolving RBB KN-R into a 0.2 mmol/L Na_2_HPO_4_ solution with a dye concentration of 0.22 mmol/L. Then, 0.88 mmol/L of DTAB was added to the wastewater and the mixture was left for 8 h to precipitate RBB KN-R. The results are presented in Figure 10A. The removal rate of RBB KN-R remained constant at over 99.0% in the pH range from 1.0 to 9.0 and then gradually decreased as the pH increased from 9.0 to 12.0. In alkaline environments (pH from 9.0 to 12.0), the sulfonate groups of the RBB KN-R molecules gradually reached complete dissociation, allowing one RBB KN-R molecule to interact with two DTAB molecules via electrostatic attraction. In this case, the dye–dye interaction was weakened, which negatively affected the formation of insoluble aggregates. Therefore, the removal rate of RBB KN-R decreased as the pH increased above 9.0.

#### 2.6.2. Effects of Salt Type and Concentration on the Removal Ratio of RBB KN-R

The salt type and concentration have been reported to have significant effects on the dye–surfactant interaction [49], so their effects on the DTAB-induced precipitation of RBB KN-R were explored in this section at an RBB KN-R concentration of 0.22 mmol/L, DTAB concentration of 0.88 mmol/L, pH 7.0, and standing time of 8 h. The selected salts included Na_2_HPO_4_, NaCl, and Na_2_SO_4_ at concentrations ranging from 0 to 1.0 mmol/L. The results in Figure 10B show that within the selected salt concentration range, Na_2_HPO_4_, NaCl, and Na_2_SO_4_ and their concentrations had no significant effect on the DTAB-induced precipitation of RBB KN-R, with the removal rate of RBB KN-R remaining at more than 99.0%. It was indicated that the DTAB-induced precipitation of RBB KN-R was insensitive to salt type and concentration.

#### 2.6.3. Effect of Standing Time on the Removal Ratio of RBB KN-R

In this section, the effect of standing time on the removal rate of RBB KN-R was studied at an RBB KN-R concentration of 0.22 mmol/L, DTAB concentration of 0.88 mmol/L, pH 7.0, and standing time from 0 to 12 h without any addition of salts. The results in Figure 10C show that as the standing time increased from 0 to 6 to 12 h, the removal rate of RBB KN-R increased from 0 to 99.5 ± 0.3% and then became constant. Thus, RBB KN-R was almost completely precipitated by DTAB in 6 h. In addition, the increase in the removal rate gradually decreased with time. This result was attributed to the gradual reduction in the RBB KN-R concentration, which decreased the opportunity for collision of the dye molecules so that the formation of the insoluble aggregates became slower.

## 3. Materials and Methods

### 3.1. Chemical Reagents

RBB KN-R and one of the surfactants (DTAB, TTAB, and CTAB) were dissolved in a solution containing Na_2_HPO_4_, NaCl, or Na_2_SO_4_ at a specified concentration. The pH of the solution was adjusted to 7.0 using 6.0 mol/L acid solutions (H_3_PO_4_, HCl, or H_2_SO_4_) and 6 mol/L NaOH solution. The concentration of RBB KN-R was maintained at 0.22 mmol/L. The purities of DTAB, TTAB, and CTAB were more than 99.0% and other chemical reagents were of analytical grade. These reagents were procured from Sinopharm Group Chemical Reagent Co. Ltd., Shanghai, China. Ultrapure water with an electrical resistance of 18.2 MΩ was employed for the preparation of solutions used in all experimental procedures.

### 3.2. Conductivity Measurements

The conductivity of the solution containing RBB KN-R and the respective surfactant (DTAB, TTAB, or CTAB) was determined after each solution stood for 1 h at a temperature of 25.0 ± 1.0 °C using a DDSJ-308A conductivity meter (Shanghai Electronic Science Instrument Co. Ltd., Shanghai, China). A reference solution consisting of only the surfactant was employed for comparison. RBB KN-R and the surfactant were dissolved in a 0.2 mmol/L Na_2_HPO_4_ solution, and the pH was adjusted to 7.0 using a 6.0 mol/L H_3_PO_4_ solution. The concentrations of the surfactants were set at 0.055, 0.11, 0.22, 0.44, 0.88, 1.32, and 1.76 mmol/L.

### 3.3. Surface Tension Measurements

The variations in surface tension with surfactant concentration in the presence or absence of RBB KN-R were evaluated by employing an automatic surface tensionmeter (QBZY-1, Shanghai Fangrui Instrument Co. Ltd., Shanghai, China) at a temperature of 25.0 ± 1.0 °C. The investigated surfactants included DTAB, TTAB, and CTAB, while the solvent used for all solutions was a 0.2 mmol/L Na_2_HPO_4_ solution with a pH of 7.0. The surfactant concentrations selected for analysis were 0.11, 0.22, 0.44, 0.66, 0.88, 1.32, and 1.76 mmol/L. All the prepared solutions stood for 1 h before the measurements.

### 3.4. UV-Vis Spectra Measurements

The UV-Vis spectra of the RBB KN-R solution and dye–surfactant mixed solution, prepared using a 0.2 mmol/L Na_2_HPO_4_ solution of pH 7.0, were examined using a UV-Vis spectrophotometer (U 3900, Hitachi, Tokyo, Japan). After each solution stood for 4 h and was then centrifuged at 10,120× *g* for 10 min, the measurements were taken at a scanning speed of 240 nm/min, a data interval of 1.0 nm, and a temperature of 25.0 ± 1.0 °C. Each spectrum obtained in the presence and absence of the surfactants (DTAB, TTAB, and CTAB) was an average of three accumulations. The surfactant concentrations ranged from 0 mmol/L to 1.76 mmol/L.

### 3.5. Molecular Dynamics Simulation

Molecular dynamics (MD) simulations were executed to explore the atomic-level interactions between RBB KN-R and three cationic surfactants (DTAB, TTAB, and CTAB) using the GROMACS 2021.5 package [50]. The initial configuration systems for the interactions RBB KN-R-DTAB, RBB KN-R-TTAB, and RBB KN-R-CTAB were generated using the PACKMOL software (version number: 20.14.2) [51]. Each simulation involved 22 dye molecules, 88 surfactant molecules, 20 Na_2_HPO_4_ molecules, and 5560 H_2_O molecules randomly placed in a cubic simulation box. The simulations were conducted at a temperature of 298.15 K and pH 7.0 to mimic experimental conditions. GAFF and SPC/E force fields were employed, with a cut-off radius of 1.2 nm for short-range van der Waals and Coulomb interactions. Long-range electrostatic interactions were computed using the Particle Mesh Ewald (PME) method. Initial equilibration of all three systems was performed for 30 ns, followed by an all-atom non-constrained MD simulation run for an additional 30 ns, utilizing a simulation time step of 5 ps, with snapshots saved at 1 ns intervals. Furthermore, density functional theory (DFT) calculations for binding energy in the RBB KN-R-DTAB, RBB KN-R-TTAB, and RBB KN-R-CTAB interactions were carried out using Gaussian software 16 (version number: Revision A.03) [52].

### 3.6. FTIR Spectra Measurements

The FTIR spectra of RBB KN-R, DTAB, a physical mixture of RBB KN-R and DTAB, and the RBB KN-R-DTAB precipitate were acquired using a Thermo Scientific Nicolet iS20 spectrophotometer (Thermo Scientific, Waltham, MA, USA). The measurements were taken employing a KBr matrix at a controlled temperature of 25.0 ± 1.0 °C. To obtain the physical mixture of RBB KN-R and DTAB, the compounds were thoroughly mixed at a molar ratio of 1:1. The RBB KN-R-DTAB precipitate was generated by allowing a 0.22 mmol/L RBB KN-R solution containing 0.88 mmol/L DTAB to stand for 24 h at 25.0 ± 1.0 °C, followed by five washes with ultrapure water. Prior to the FTIR measurements, all the four samples were subjected to drying in a DZF-6032 vacuum-drying oven (Shanghai Yiheng Scientific Instruments Co. Ltd., Shanghai, China) at 100 °C for 48 h.

### 3.7. Determination of Removal Ratio of RBB KN-R

Removal ratio of RBB KN-R (*R*_RBB_) was evaluated as Equation (2).

(2) $$R_{RBB}=\frac{C_{0}-C_{r}}{C_{0}}\times 100\%$$

where *C*_0_ represents the chroma value of the initial RBB KN-R-surfactant solution and *C_r_* represents the chroma value of the residual solution following precipitation. The measurement of each chroma value was performed using a chroma instrument (CN60M/SD9011, Qiangdao Westmid Technology Co. Ltd., Qingdao, China) subsequent to centrifugation of each solution at 10,200× *g* for 10 min. Before measurements, the instrument was calibrated with platinum–cobaltic standard solutions at chroma values of 0, 50, 100, 250, and 500 [53]. The chroma value of 1 was defined as the color produced when there were 2 mg of hexaaquacobalt (IV) chloride hexahydrate and 1 mg of platinum (in the form of hexachloroplatinic (IV) acid) in each solution.

### 3.8. Statistical Analysis

In this work, each experiment was performed in a minimum of three replicates. Statistical analysis was conducted using Microsoft Office Professional Plus 2013 (version number: 15.0.5589.1001) employing one-way analysis of variance, with a significance level set at *p* ≤ 0.05 to indicate statistical significance. The results are presented as mean values ± standard deviation.

## 4. Conclusions

To establish a surfactant-based methodology conducive to the efficient removal of RBB KN-R from wastewater, we delved into the examination of interactions between RBB KN-R and three cationic surfactants possessing identical head groups but varying alkyl chain lengths (DTAB, TTAB, and CTAB). Our empirical findings delineate the formation of a dye–surfactant complex in a 1:1 molar ratio between RBB KN-R and each surfactant, primarily driven by electrostatic attraction. Moreover, it is observed that the electrostatic affinity intensifies with an augmentation in alkyl chain length. The resultant dye–surfactant complex exhibits superior surface activity compared to individual surfactants, enabling interactions through hydrophobic forces to manifest dye–surfactant aggregates when the molar ratio is less than 1:1. Within these mixed aggregates, the self-assembly of RBB KN-R molecules gives rise to the formation of dye aggregates. Because the increase in alkyl chain length improved the surfactant hydrophobicity, TTAB and CTAB could encapsulate the dye aggregates in the interior of the mixed aggregates, but DTAB fails to achieve this encapsulation. The spatial distribution of the RBB KN-R aggregates on the surface of the RBB KN-R-DTAB mixed aggregates leads to diminished stability. Consequently, at a DTAB concentration lower than CMC, insoluble particles are readily formed and separated from surfactant aggregates at an RBB KN-R and DTAB molar ratio of 1:4. At this critical ratio, the entire RBB KN-R precipitates from the dye–surfactant mixed solution, with only 7.5 ± 0.5% of DTAB retained in the precipitate. We further investigated the effects of pH, salt type and concentration, and standing time on the removal rate of RBB KN-R. Our results demonstrated that RBB KN-R could be removed with an efficiency nearby 100% within a pH range of 1.0 to 9.0 and a standing time of 6 h. Furthermore, the presence of different salt types and concentrations did not significantly affect the precipitation process. Thus, this strategic approach accomplishes the concurrent successful removal of RBB KN-R and the efficient separation of RBB KN-R from DTAB, presenting a significant advancement in the economically viable eradication of dyes from wastewater.

## Figures and Tables

**Table 1:** The values of binding constant for RBB KN-R -DTAB, TTAB, and CTAB systems.

Surfactant	Binding Constant (*k*_b_, L/mol)	Linear Correlation Coefficient (*R*^2^)
DTAB	310	0.9995
TTAB	350	0.99958
CTAB	490	0.99817

## Data Availability

The data are contained within the article.
